# Systemic Sarcoidosis With Neurosarcoidosis Features as a Risk Factor for Multifocal Osteonecrosis

**DOI:** 10.7759/cureus.66791

**Published:** 2024-08-13

**Authors:** Hee Jae Jung, Jamal Mikdashi

**Affiliations:** 1 Internal Medicine, University of Maryland Medical Center, Baltimore, USA; 2 Rheumatology, University of Maryland School of Medicine, Baltimore, USA

**Keywords:** rheumatic disorders, neurosarcoidosis, multifocal osteonecrosis, osteonecrosis, sarcoidosis

## Abstract

Sarcoidosis is a systemic inflammatory disease that affects diverse organs such as the lungs, skin, eyes, and brain. Osseous involvement in sarcoidosis usually affects bones of the appendages with direct infiltration of non-caseating granulomas without bony infarcts. Symptoms of sarcoid bone lesions respond well to corticosteroid therapy. In contrast, corticosteroids act as a risk factor for the development of osteonecrosis resulting in pain and disability. Osteonecrosis that involves three or more different anatomic sites, defined as multifocal osteonecrosis (MFON), is rare. MFON has not been documented in the setting of sarcoidosis. We report a systemic sarcoidosis patient with predominant neuropsychiatric manifestations, who progressively developed MFON. Despite the limited use of corticosteroid treatment, the high burden of systemic sarcoidosis and its related neuropsychiatric involvementmay have collectively contributed to the development of MFON. This case highlights the rare association of MFON with systemic sarcoidosis and the need for further investigation into the underlying pathogenesis of MFON to prevent disability and morbidity.

## Introduction

Sarcoidosis is a systemic disease characterized by T lymphocyte-mediated noncaseating granuloma formation, most commonly affecting the lungs, eyes, and skin. Osseous involvement in sarcoidosis mainly affects the hands or feet, and in rare cases, the axial skeleton or the skull [1]. Most bone involvement remains asymptomatic and is usually found incidentally [2]. Punched-out, permeative, or reticular changes can be seen in conventional radiographic imaging [3]. Spinal and axial involvements often present with back pain with sclerotic lesions of vertebral bodies or pedicles on conventional radiographs [4]. Pathologically, these lesions show sarcoid granuloma formation [2]. Treatment of osseous involvement is not well-defined, but corticosteroids are often used as patients with osseous involvement often have sarcoidosis in other organs [1]. Osteopenia and osteoporosis may develop due to a combination of factors such as treatment with corticosteroids and the systemic inflammatory state of the disease. Multifocal osteonecrosis (MFON) has not been reported in the English literature in association with sarcoidosis, to the best of our knowledge.

Osteonecrosis is characterized by the death of bone tissue due to impaired subchondral blood supply caused by various mechanisms and commonly affects the epiphysis of long bones at weight-bearing joints like the hips or knees. MFON is defined as osteonecrosis involving three or more different anatomical sites. For example, knees, hips, and shoulders would be three different anatomical sites; bilateral knees and a hip joint would only be considered two different anatomical sites [5]. MFON comprises approximately 3-5% of all osteonecrosis cases, and most commonly affects hips, knees, and shoulders and progresses in that order with bilateral involvement [5,6].

Corticosteroid use is recognized as the leading risk factor for non-traumatic osteonecrosis [7]. However, the extent to which corticosteroids contribute to the pathogenesis of MFON is not fully understood. While many rheumatological diseases, such as systemic lupus erythematosus (SLE) and Sjögren’s syndrome, have been identified as risk factors for osteonecrosis, there have been no available data in association with sarcoidosis. Vertebral osteonecrosis involving the vertebral body has been suggested in one report [8].

Diagnosis of MFON is challenging and requires a high index of suspicion for early detection. Most patients do not display symptoms until the disease has progressed. The recommended modality of diagnosis for MFON is magnetic resonance imaging (MRI) [7]. However, the limitations in cost and time make it difficult to utilize this tool for early diagnosis of MFON, leading to debilitating arthropathies and subsequent joint replacement surgeries.

In this article, we report a unique case of MFON in a systemic sarcoidosis patient with predominant neuropsychiatric features.

## Case presentation

A 44-year-old female presented to our clinic with pain in her shoulders, knees, and hip joints. The patient has MRI-proven osteonecrosis in both distal femurs, both femoral heads, and the head of the right humerus. She was treated with supportive care and pain management with oral medications and physical therapy, and eventually received total joint replacement surgery in the right knee, hips, and right shoulder.

The patient was diagnosed with pulmonary sarcoidosis at age 30, confirmed by biopsy. Pulmonary sarcoidosis was treated with short-term oral corticosteroids with adequate response and resolution of pneumonitis and adenopathy. Shortly after diagnosis, the patient presented with ocular symptoms requiring only topical corticosteroid therapy for a month with adequate response.

**Figure 1:**
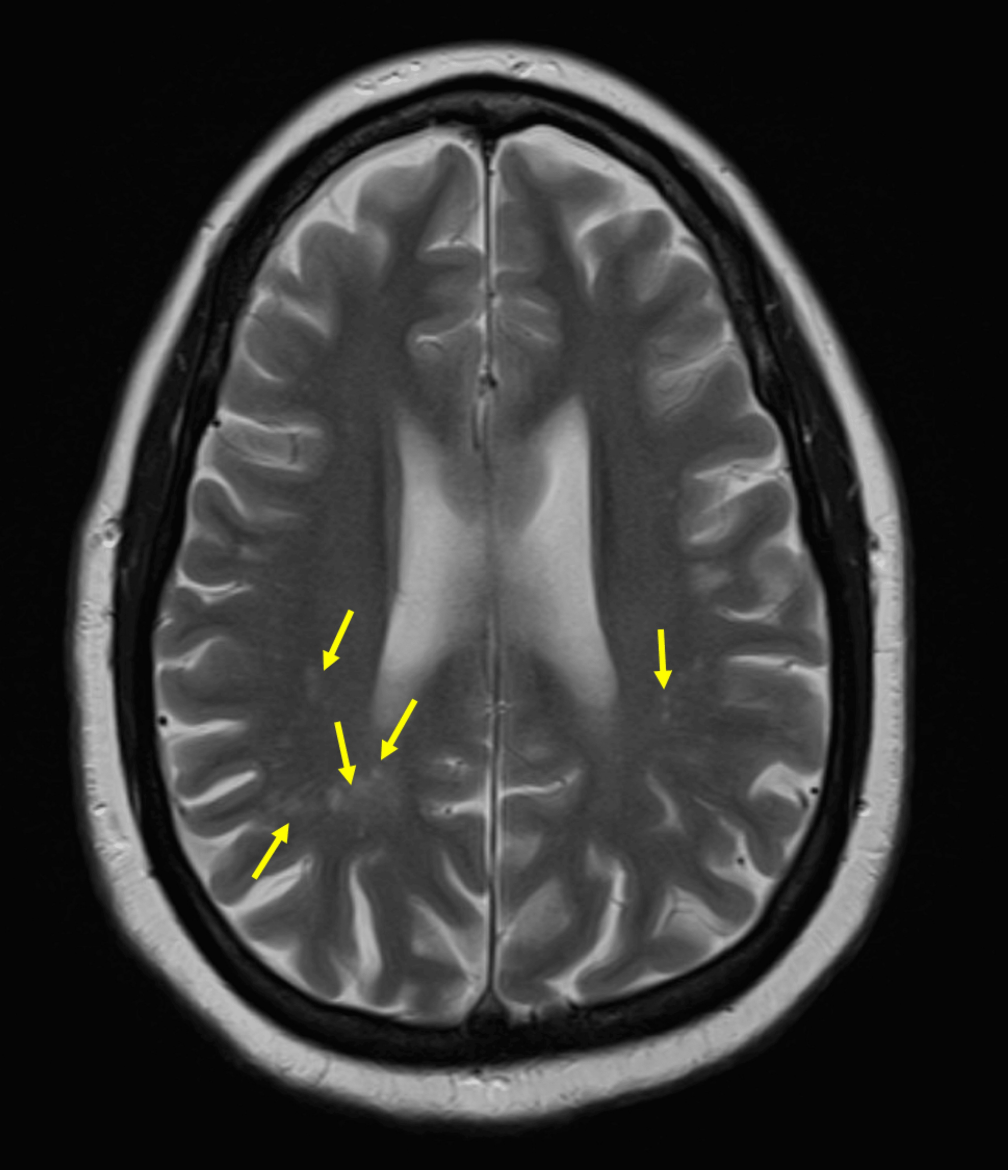
Multiple subcortical white matter lesions. Note the multiple subcortical white matter hyperintense lesions (arrowheads) on the T2-weighted image of brain MRI without contrast.

**Figure 2:**
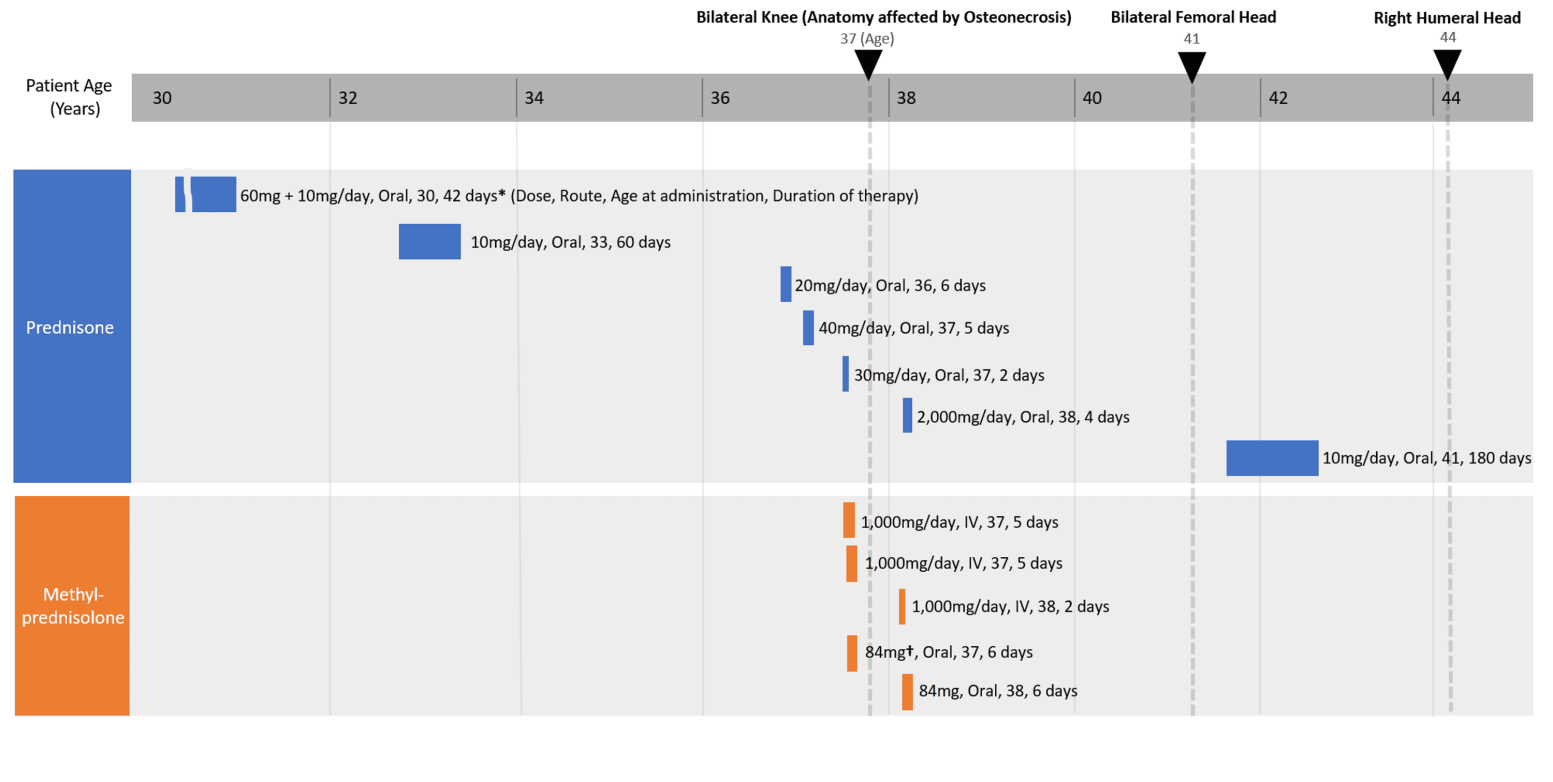
Corticosteroid dose and duration by patient age. Upside-down triangles (▼) depict the initial discovery of osteonecrosis by radiological imaging in the respective joints. Bilateral knees and right humeral head osteonecrosis were initially diagnosed with MRI imaging. Bilateral femoral head osteonecrosis was initially discovered with abdomen/pelvis CT and later confirmed with MRI imaging. The above depicts the initial discovery by CT for bilateral femoral head osteonecrosis. ^*^ The patient initially received a max dose of 60 mg of prednisone and tapered over six weeks, which amounted to a total of approximately 900 mg. The duration of therapy could not be identified due to a lack of medical records. ^†^ Oral 84 mg methylprednisolone depicts one methylprednisolone 4 mg taper pack dose.

The patient's course was complicated by neurosarcoidosis. At around age 37, the patient started to develop a clinical syndrome featuring multiple seizures, headaches, speech disturbances, cognitive dysfunction, and behavioral challenges with psychosis leading to multiple hospital admissions. Initial brain MRI revealed multifocal subcortical white matter lesions that were non-enhancing with gadolinium contrast (Figure 1). Multiple CSF studies and repeated neuroimaging studies yielded no clear evidence for infection, malignancy, or demyelinating syndrome (Table 1). Thus, the diagnosis of neurosarcoidosis was presumed most likely given the patient's prior diagnosis of pulmonary sarcoidosis. The neuropsychiatric conditions were addressed with short-term high-dose corticosteroids (Figure 2). The patient’s neuropsychiatric symptoms improved with corticosteroid treatment. However, the patient continued to have mild speech and cognitive dysfunctions that remained unchanged throughout her follow-ups.

**Table 1:** Multiple CSF studies done at age 37.

CSF	Reference range	CSF study 1	CSF study 2	CSF study 3
Glucose	40 - 70 mg/dL	78		57
Protein	12 - 60 mg/dL	32	29	25
RBC	0 - 0/mcL	2	48	6
WBC	0 - 5/mcL	2	1	1
Polymorphs %		0	7	0
Lymphocytes %		46	66	86
Monocytes %		54	27	14
CSF clarity		Clear	Clear	Clear
CSF color		Colorless	Colorless	Colorless
IgG	0.0 - 8.6 mg/dL		2.3	2.6
Albumin	11 - 48 mg/dL		8	12
IgG Index	0.0 - 0.7		0.7	0.5
Angiotensin-converting enzyme (ACE) level	0.0 - 2.5 u/L		<0.4	
IgG/albumin ratio	0.00 - 0.25		0.29	0.22
Oligoclonal bands		None	None	None
Beta 2 microglobulin				None
CSF gram stain and culture		Negative	Negative	

The patient suffered two intravascular thrombotic events. Once as an unprovoked left cephalic vein thrombosis at age 34. This was managed with short-term anticoagulation therapy with warfarin.

**Figure 3:**
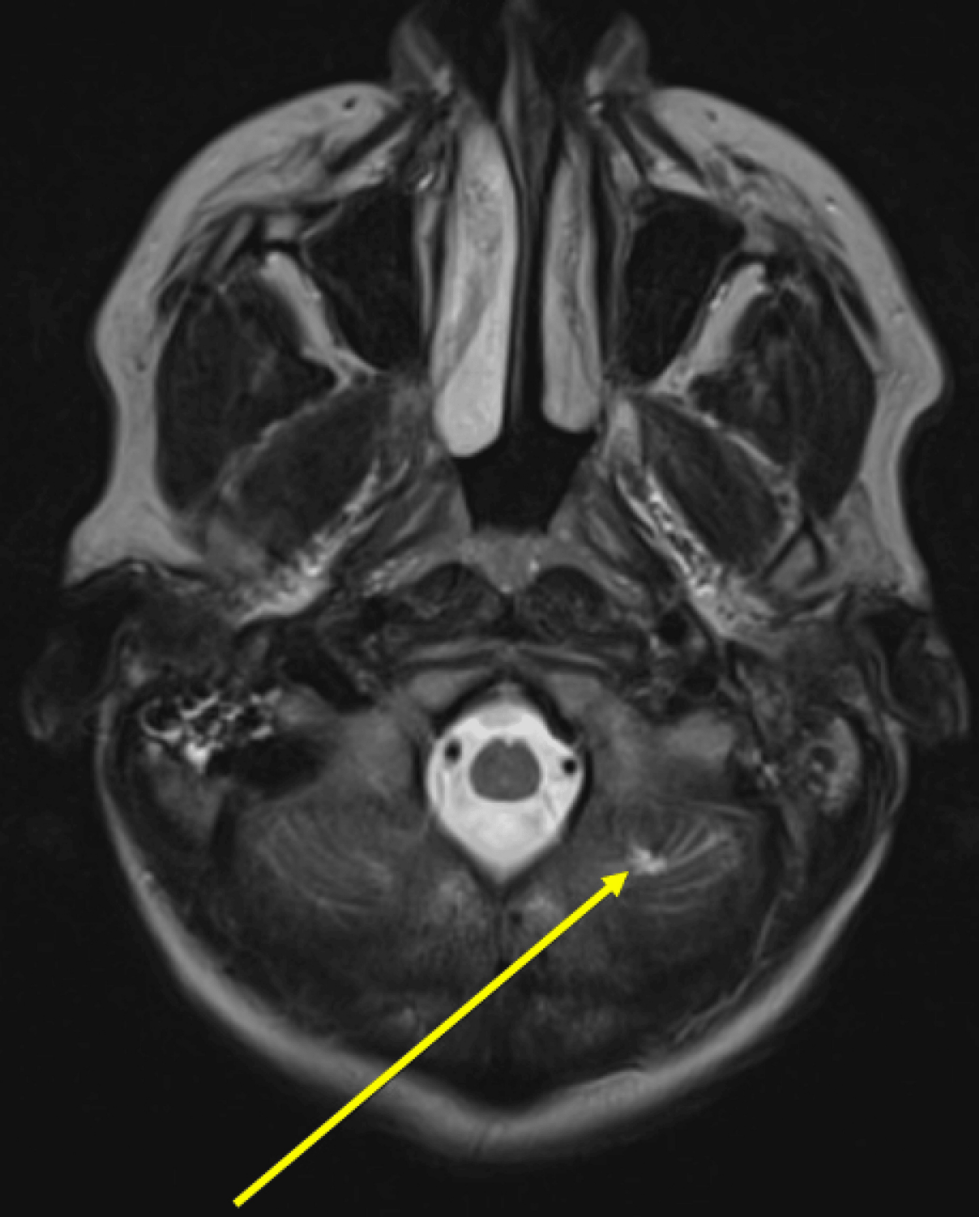
Silent cerebellar infarct discovered at age 43. Note the chronic lacunar infarct in the left cerebellar hemisphere (arrowhead) on the T2-weighted image of the brain MRI.

The second event was a silent left cerebellar infarct, incidentally discovered in a follow-up brain MRI at age 43 (Figure 3) that was not present on brain MRI from age 39. Other causes of ischemic stroke were investigated after the incidental discovery of cerebellar infarct on brain imaging. She was negative for sickle cell trait or disease, antiphospholipid antibodies, protein C deficiency, protein S deficiency, and anti-thrombin III deficiency. Other autoimmune disease workups were negative (Table 2). The patient was negative for HIV and syphilis infections. Glycosylated hemoglobin (HbA1c, 5.2%), thyroid-stimulating hormone (TSH), free T4 (fT4), and lipid panel were normal (Table 3). Other routine laboratory workups were non-significant.

**Table 2:** Autoimmune disease workup. dsDNA: double-stranded DNA; ANA: antinuclear antibody; IFA: immunofluorescence assay.

Autoantibodies	Reference range	Results
dsDNA antibody	0 - 9 IU/mL	<1
ANA direct	Negative	Negative
ANA IFA		Negative
Rheumatoid factor	0.0 - 13.9 IU/mL	11.1
Cyclic citrullinated peptide IgG/IgA	0 - 19 ZZ	7

**Table 3:** Lipid panel. HDL: high-density lipoprotein; LDL: low-density lipoprotein; VLDL: very low-density lipoprotein.

	Reference range	Results
Cholesterol	≤199 mg/dL	161
Triglycerides	≤150 mg/dL	76
HDL	32 - 83 mg/dL	55
LDL	≤100 mg/dL	91
VLDL	5 - 40 mg/dL	15

**Figure 4:**
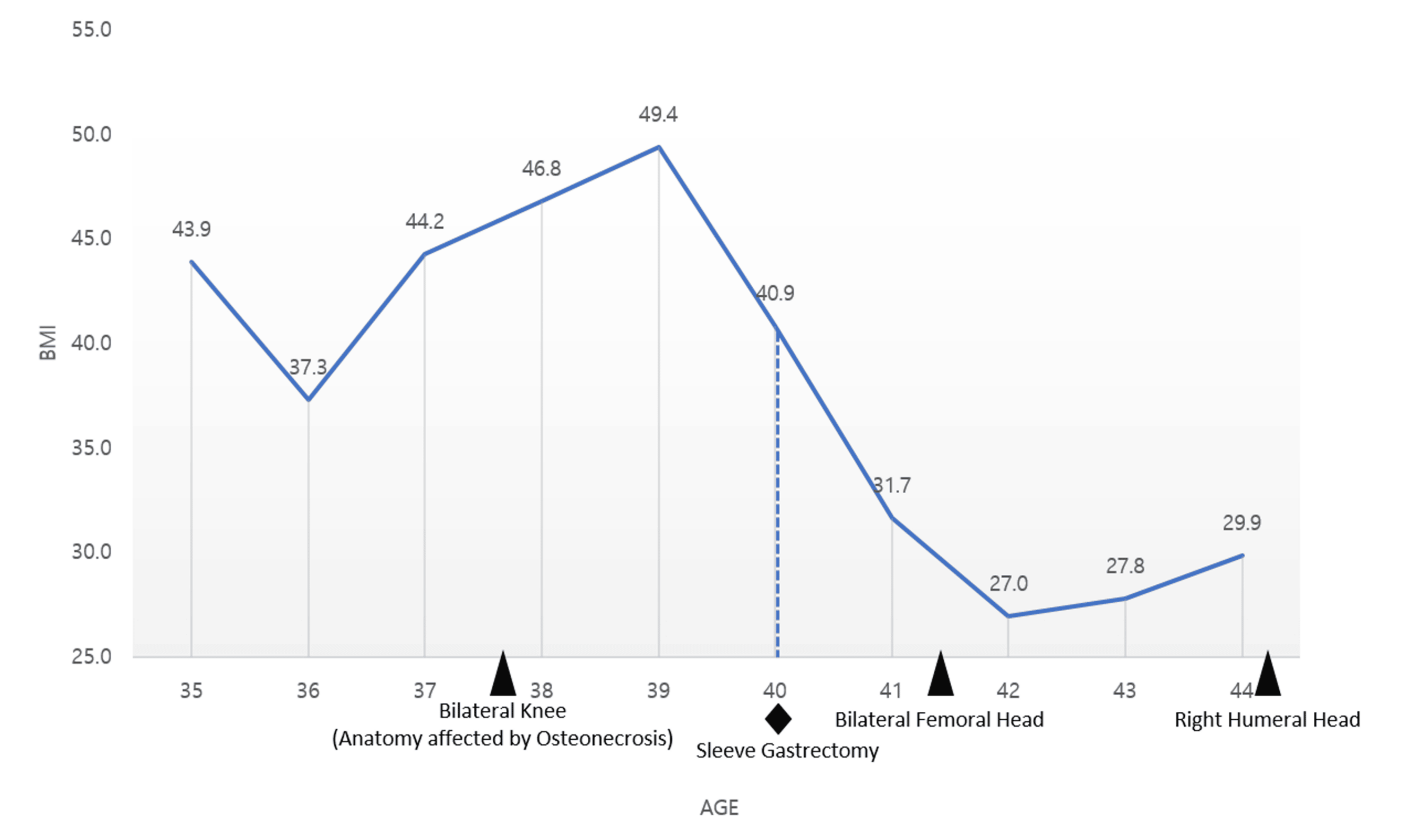
Change in BMI by patient age. Black triangles depict the initial discovery of osteonecrosis by radiological imaging in the respective joints. Diamond depicts the age when the patient received sleeve gastrectomy.

The patient also had a history of morbid obesity for which she underwent sleeve gastrectomy at the age of 40. She was able to lose weight from a BMI of 49.4 (at age 30) to 29.9 (at age 44) on her most recent presentation to our clinic (Figure 4).

Joint disease and osteonecrosis

**Figure 5:**
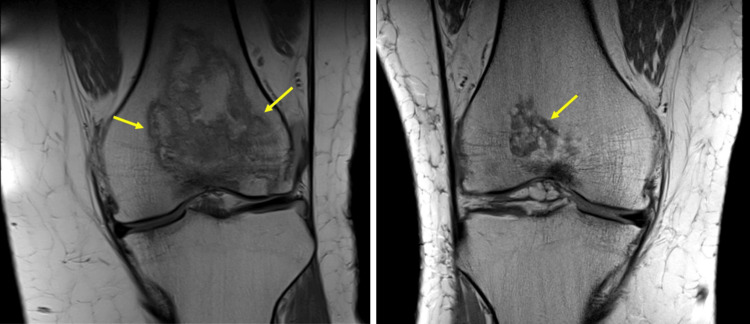
Bilateral knee osteonecrosis. Large bony infarcts are evident in both images. Note the serpentine low-signal intensity bands (arrowheads) surrounding the central area of necrosis on bilateral knee T1-weighted MRI.

The patient had a chronic history of multijoint arthralgias (knees, wrist, shoulder) since age 33 that was poorly controlled with oral analgesics and nerve blocks. By age 36, the patient's knee arthralgia progressed, leading to knee instability and falls. MRI of both knees showed multifocal joint infarcts (Figure 5), which led to bilateral knee chondroplasty and right knee replacement at age 38.

**Figure 6:**
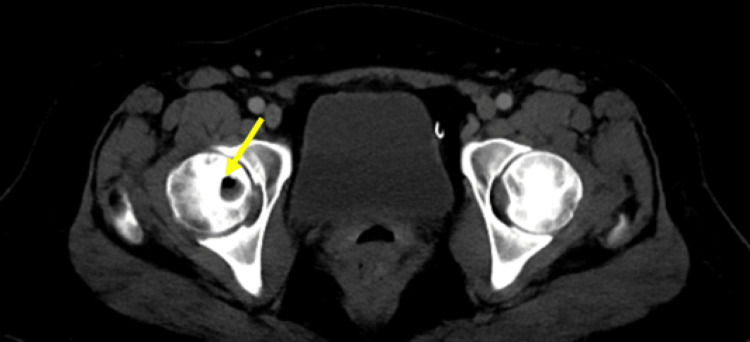
Femoral head osteonecrosis on CT. Earliest detection of avascular necrosis of the femoral head in abdomen/pelvis CT scan. Note the large necrosis in the head of the right femur (arrowhead).

**Figure 7:**
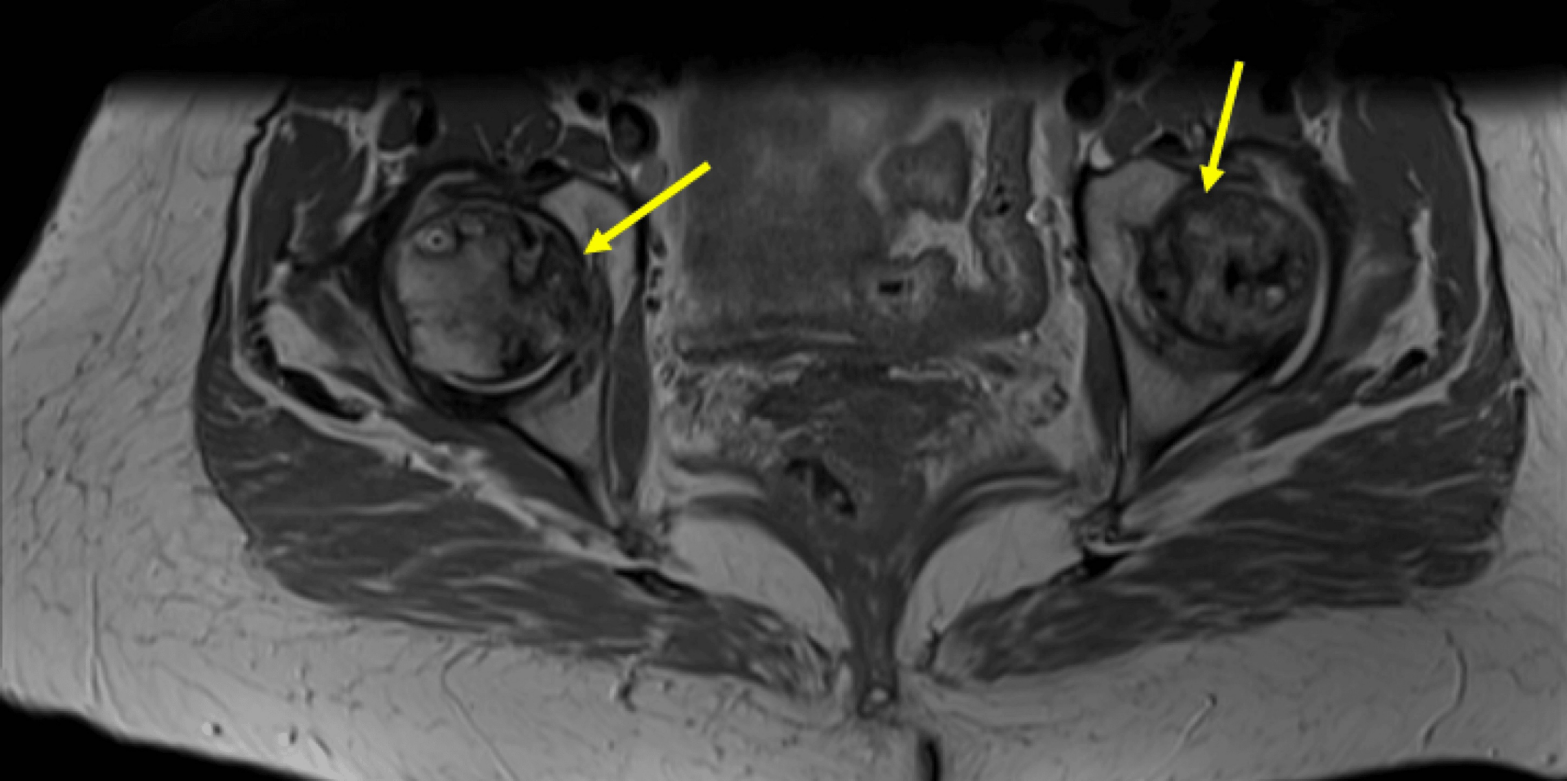
Bilateral femoral head osteonecrosis on MRI. Osteonecrosis of bilateral femoral heads in T1-weighted MRI images of the pelvis. Note the serpentine low-signal intensity bands (arrowheads) surrounding the central area of necrosis.

Bilateral osteonecrosis changes were discovered incidentally on CT of the abdomen and pelvis, taken as part of an evaluation of abdominal pain (Figure 6) at age 41. However, this was not reported in the radiology read at the time. The patient continued to endorse multijoint pain but started to develop worsening left leg pain that warranted further evaluation. MRI imaging of bilateral hips revealed extensive avascular necrosis and collapse of femoral heads (Figure 7). Subsequently, the patient received a left total hip replacement at age 42.

**Figure 8:**
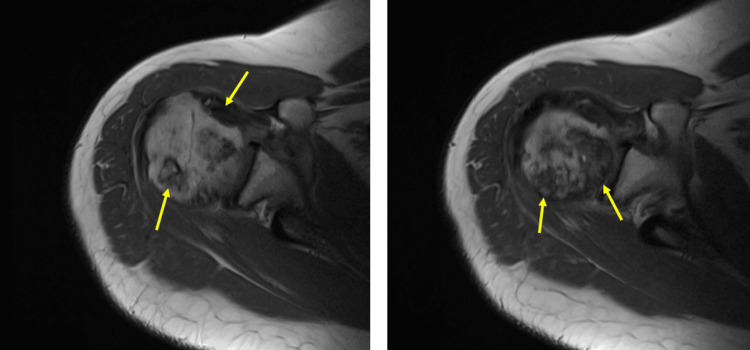
Humeral head osteonecrosis. Osteonecrosis of the humeral head in T1-weighted MRI image of the right shoulder. Note the serpentine low-signal intensity bands (arrowheads) surrounding the central area of necrosis.

At age 44, the patient developed progressive right shoulder pain. MRI imaging revealed evidence of posterior medial humeral head osteonecrosis and advanced degenerative changes at the glenohumeral joint (Figure 8). The patient underwent a right shoulder joint replacement.

Surgical pathology

Pathology reports generated after respective joint arthroplasties were reviewed. Knee pathology reported that the bone marrow showed focally increased plasma cells, lymphocytes, and foamy cells. The lymphocytes were composed predominantly of CD3+ T cells with scant CD20+ B cells and numerous plasma cells. Femoral head pathologies reported changes consistent with avascular necrosis (bony trabeculae with empty lacunae, fat necrosis, intertrabecular fibrosis, and focal reactive bone). No granuloma or giant cells were detected. Right humeral head pathology showed reactive changes and focal osteonecrosis.

Social history

The patient reported a tobacco use history of 0.4 packs/day for the past 20 years. The patient had stopped drinking alcohol years ago before the initial presentation at our clinic.

Family history

The patient reported hypertension, anxiety disorder, type 2 diabetes mellitus, asthma, stroke, and ischemic heart disease in her mother. Otherwise, the family history was noncontributory.

## Discussion

Osteonecrosis is thought to be caused by a combination of factors leading to localized ischemia [7]. Although a variety of causes, including corticosteroids and various rheumatologic diseases, have been identified as risk factors, sarcoidosis has not been yet investigated. Osseous involvement of sarcoidosis is typically present in the hands, feet, and axial skeletons with direct infiltration of the bone by granulomas. Osteonecrosis is not a well-known feature of sarcoidosis and there has been only one case report of osteonecrosis associated with sarcoidosis in the vertebral body [8]. MFON with rapid progression to joint collapse is not reported in association with sarcoidosis. The progressive involvement of large joints suggests an underlying sarcoid-related pathogenesis that should be further investigated, especially given the high burden of neurosarcoidosis in this patient.

As sarcoidosis is a systemic disease, it is also known to affect vasculature. Some studies have shown evidence of vasculopathy with endothelial damage and inflammation in sarcoidosis patients [9,10]. Disruption of endothelial function and inflammation can cause thrombosis and lead to ischemia, which might have been a contributing factor for MFON in this patient. This is further supported by the fact that the patient endured at least two thromboembolic events (left arm superficial vein thrombosis and left cerebellar infarct) throughout the course of her disease. It is also postulated that cerebrovascular events in neurosarcoidosis patients are associated with vasculopathy [11,12]. It is important to note that the patient had signs of neurosarcoidosis and a thrombotic event very early in the course, outlining the heavy burden of the disease. Multi-organ involvement of sarcoidosis, including the CNS, is a sign of severity and heavy burden of sarcoidosis. In that context, it is possible that the severity of sarcoidosis contributed highly to the development of MFON in this patient.

The intermittent use of corticosteroids should also be considered in this patient, as it is one of the most common known risk factors for MFON (Table 4) [5,13-28]. In our case, however, corticosteroid use alone cannot account for the multiple large joint involvement without any other reported significant risk factors.

**Table 4:** Risk factors for the development of multifocal osteonecrosis.

Risk factors for the development of multifocal osteonecrosis	
Medications	Corticosteroids, protease inhibitors
Alcohol use disorder	Chronic alcohol ingestion
Malignancy	Acute lymphocytic leukemia, non-Hodgkin’s disease
Connective tissue diseases	Systemic lupus erythematosus, antiphospholipid syndrome, dermatomyositis/polymyositis, systemic sclerosis, Sjogren’s syndrome
Infection	Human immunodeficiency virus
Coagulopathies	Antithrombin III deficiency, protein S deficiency, factor V Leiden gene mutation, factor VIII related X-linked thrombophilia, G20210A prothrombin gene heterozygosity
Occupational hazards	Chronic aluminum exposure
Gastrointestinal disorders	Inflammatory bowel diseases, especially those with prior arthropathies and osteoporosis and in severe diseases
Hemoglobinopathy	Sickle cell disease
Trauma	Pancreatic trauma
Organ transplant	Renal transplantation
Neurologic disorders	Multiple sclerosis

The dose and duration of corticosteroid use that causes osteonecrosis is still in debate. One study reported that a total dosage of prednisone ranging from 1,800 to 15,505 mg within a mean period of 5.3 months (range: one to 16 months) from initiation of steroid therapy to diagnosis was associated with corticosteroid-induced osteonecrosis [29]. There is no safe dose of corticosteroids that induces osteonecrosis, but the longer duration of usage and higher dose may pose a higher risk [30]. According to the Association Research Circulation Osseous (ARCO) task force, classification criteria of corticosteroid-associated osteonecrosis of the femoral head requires a history of corticosteroid use of more than 2,000 mg of prednisolone or its equivalent within three months, diagnosis of osteonecrosis within two years after steroid usage and excluding any other etiologies [31].

Although the patient received various corticosteroid therapies throughout the clinical course, it is not sufficient to explain the involvement of multiple joints. As seen in Figure 2, short-term, high-dose methylprednisolone (1,000 mg/day for a total of 10 days or 10,000 mg total intravenously within one month) may have contributed to the pathogenesis of bilateral knee osteonecrosis. However, the fact that the onset of severe knee joint pain and gait abnormalities preceded the patient's short-term, high-dose corticosteroid regimen, suggests that joint damage may have occurred before steroid therapy. The time to confirmation of bilateral femoral head avascular necrosis and humeral head osteonecrosis by imaging since the use of high-dose corticosteroids is also longer than the ARCO task force criteria. There were two instances of CT imaging in our patient between age 37 and year 41 of the abdomen and pelvis, which did not reveal any abnormalities in the hip. It must be considered though that CT is not the most sensitive modality [7], and the CT scans were not targeted to screen for joint abnormalities.

Angiogenesis and increased intraosseous pressure are proposed as one of the mechanisms of corticosteroid-induced osteonecrosis [7]. The patient's history of obesity may have contributed to the pathogenesis of bilateral knee osteonecrosis, but it alone would not explain the involvement of bilateral femoral heads and right humeral head as the clinical syndrome presented after the patient’s bariatric surgery and subsequent weight loss (Figure 4).

## Conclusions

Many autoimmune disorders are considered a risk for inciting osteonecrosis, but sarcoidosis has not been well documented. The authors speculate that systemic sarcoidosis is associated with a high burden of parenchymal disease and vasculopathy may lead to endothelial dysfunction and expedite corticosteroid-mediated osteonecrosis. Progressive multiple joint involvements in the setting of systemic sarcoidosis with predominant neurosarcoidosis features are not yet reported and warrant further investigation. Clinical vigilance in high-risk patients and the application of screening methods of X-ray examination, joint MRI with short-term inversion recovery, or radionuclide scans of specific joints are required to determine early detection of osteonecrosis.
